# Associations Between Cessation of Second-Line Therapies and Relapse Rates of Childhood Refractory Minimal-Change Nephrotic Syndrome: A Single-Center, Retrospective Chart Review

**DOI:** 10.1016/j.curtheres.2022.100671

**Published:** 2022-04-12

**Authors:** Jing Jin, Yufeng Li, Yaju Zhu, Jiajia Ni

**Affiliations:** Department of Pediatric Nephrology, Rheumatology, and Immunology, Xinhua Hospital Affiliated to Shanghai Jiaotong University School of Medicine, Shanghai, China

**Keywords:** children, immunosuppressant, minimal-change disease, nephrotic syndrome, steroid-dependent nephrotic syndrome, steroid-resistant nephrotic syndrome

## Abstract

**Background:**

Most patients (≥85%) with minimal-change nephrotic syndrome (MCNS) respond to corticosteroid treatment. However, about 10% to 20% of patients with MCNS have steroid-resistant nephrotic syndrome and 25% to 43% of patients have steroid-dependent nephrotic syndrome or frequent-relapse steroid-sensitive nephrotic syndrome. Patients with refractory MCNS are treated with various second-line therapies.

**Objectives:**

This study aimed to evaluate the associations between the use of various second-line therapies and relapse rates in Chinese patients with childhood refractory MCNS.

**Methods:**

In this study, patients with childhood nephrotic syndrome renal biopsy proved to be “minimal change” from a single tertiary-care center between January 2002 and July 2018 were identified. A Total of 56 medical charts of patients treated with 1 of these second-line immunosuppressors: cyclophosphamide (CYC), mycophenolate mofetil (MMF), or tacrolimus (TAC) were reviewed. Patients were divided into CYC (n = 24), MMF (n = 20), and TAC (n = 12) groups according to the second-line therapy administered. Baseline characteristics, immune status, immunocomplex deposition in the renal tissue, and treatment outcomes were analyzed.

**Results:**

The ratio of patients with steroid-resistant nephrotic syndrome and steroid-dependent nephrotic syndrome in the CYC, MMF, and TAC groups did not differ significantly (*P* = 0.721). The immunofluorescence assay did not show any significant differences in immunocomplex deposition identified in renal biopsy specimens among the 3 groups. The rate of steroid-free remission in the TAC group (75%) was higher than that in the MMF (55%) and CYC (25%) groups (*P* = 0.012). At the last follow-up, two-thirds of children in the TAC group had a relapse following discontinuation of therapy. In the TAC group, patients for whom steroids were withdrawn had significantly higher levels of immunoglobulin G at the onset of nephrotic syndrome than those for whom steroids were continued (*P* = 0.017). In the MMF group, children with relapse had a significantly higher percentage of CD16^+^CD56^+^-positive cells than those without relapse (*P* = 0.042). The relapse rate after treatment discontinuation was significantly different among the 3 groups (*P* = 0.035). Notably, the relapse rate after treatment discontinuation in the CYC group was lower than those in the other 2 groups (*P* = 0.035).

**Conclusions:**

In this small population of Chinese patients with childhood refractory MCNS, the relapse rate following TAC therapy was higher than that following MMF or CYC therapy. Different proportions of CD16^+^CD56^+^-positive cells might be associated with relapse rates in patients with MCNS receiving MMF treatment. (*Curr Ther Res Clin Exp*. 2022; 83:XXX–XXX)

## Introduction

Nephrotic syndrome (NS) is the most common form of glomerular disease in pediatric patients. Idiopathic NS, also known as minimal-change nephrotic syndrome (MCNS), is a glomerular disease characterized by massive proteinuria, hypoalbuminemia, edema, and hyperlipidemia and accounts for 77% of all NS cases in children.^1^ Most patients with MCNS show a good response to steroid treatment and have a favorable long-term prognosis. Despite this, 70% of pediatric patients with steroid-sensitive NS (SSNS) who show good response, relapse and develop frequently relapsing SSNS (FR-SSNS) or steroid-dependent NS (SDNS).^2^ Furthermore, a small proportion of patients present steroid-resistant NS (SRNS) and have a poor prognosis, with approximately 50% developing end-stage renal disease within 10 years.^3^^,^^4^

Alternative treatments such as second-line immunosuppressants and immunological interventions are often prescribed for the refractory forms of NS, including SRNS, SDNS, and FR-SSNS.^5^ Second-line immunosuppressants, including cyclophosphamide (CYC), mycophenolate motefil (MMF), cyclosporine A, and tacrolimus (TAC), are used to prevent severe adverse effects associated with prolonged steroid exposure.^6^ Intravenous pulse CYC is primarily used to treat steroid-dependent and frequently relapsing MCNS,^7^ MMF is used for the treatment of SDNS, whereas cyclosporine A and TAC, a calcineurin inhibitor, are often used as second-line therapies for SRNS in pediatric patients.^5^, ^6^, ^7^, ^8^, ^9^, ^10^, ^11^

Several studies have indicated that immune cells and immunoglobulins (IGs) may be associated with remission and relapse rates in pediatric primary NS.^12^, ^13^, ^14^ Hence, an in-depth analysis of the effectiveness of immunosuppressants for the treatment for pediatric NS is required.

The aim of this study was to evaluate the influence of second-line immunosuppressive therapies on the relapse rates of pediatric refractory NS. This retrospective study included pediatric patients with refractory NS treated with TAC, MMF, or CYC. Herein, we report our single-center experience with second-line immunosuppressive therapy for pediatric refractory MCNS.

## Patients and Methods

The study included patients younger than age 18 years who underwent treatment at a single tertiary care center between January 2002 and July 2018. All pediatric patients who had undergone a renal biopsy were searched in our hospital's electronic medical database using the search terms *minimal change* and *nephrotic syndrome*. Records of each patient with a diagnosis of MCNS were evaluated and matched with the patient's records in the renal biopsy database. Many patients with MCNS were found to have been treated with *Tripterygium wilfordii* (a Chinese traditional medicine) tablets as second-line immunosuppressive therapy. Hence, we added another inclusion criteria: “treatment with second-line immunosuppressants such as CYC, MMF, and TAC” to limit our results to standard approved therapies. Children with MCNS who received *Tripterygium wilfordii* as a second-line immunosuppressant and 1 patient treated with MMF combined with TAC were excluded from the study.

The inclusion criteria were primary NS; biopsy-proven MCNS; SRNS, SDNS, or FR-SSNS refractory to steroid therapy; and treatment with second-line immunosuppressants such as CYC, MMF, and TAC. Patients with infantile (or congenital) NS, secondary NS, glomerulonephritis, or systemic diseases were excluded from the study. Patients’ data were extracted after reviewing their medical histories. Data on the duration of various drug therapies and response rate to each drug were also collected. The response to treatment in patients who completed ≥3 months of therapy was recorded.

### Categories of refractory MCNS

Refractory NS is composed of SRNS, SDNS, and FR-SSNS. SSNS is characterized by the achievement of complete remission within the initial 4 weeks of corticosteroid therapy.^15^ SDNS falls under the subcategory of SSNS and is defined as 2 consecutive relapses during corticosteroid therapy or within 14 days of discontinuation of therapy.^15^ FR-SSNS, also a subcategory of SSNS, is characterized by patients having 2 or more relapses within 6 months of the initial response, or 4 or more relapses in any 12-month period.^15^ SRNS is characterized by failure to achieve complete remission after 4 weeks of corticosteroid therapy.^15^

Complete remission was defined as urine protein to creatinine ratio (uPCR) <200 mg/g (<20 mg/mmol) or <1+ of protein on urine dipstick for 3 consecutive days.^15^ Partial remission was defined as a decrease in proteinuria by ≥50% from that observed at the time of presentation and absolute uPCR between 200 and 2000 mg/g (20–200 mg/mmol).^15^ No remission was defined as failure to achieve a 50% decrease in proteinuria from baseline or persistent excretion uPCR >2000 mg/g (>200 mg/mmol).^15^ Steroid-free remission was defined as the achievement of complete remission for more than 14 days after discontinuation of therapy.

### Immunocomplex deposition assessed by immunofluorescence

MCNS was divided into 5 subtypes based on the intensity of immunofluorescence staining of the renal tissue: major IgM deposition indicated by strong positive (++) staining for IgM; IgG + A + M + complement deposition indicated by positive staining for IgG, IgA, IgM, and complement proteins; IgG + A + M deposition indicated by identical intensity of immunofluorescence staining for IgG, IgA, and IgM; major complement deposition indicated by positive staining only for complement proteins and not for IgG, IgA, and IgM; and no immunocomplex deposition indicated by no immunofluorescence staining with antibodies against IgG, IgA, IgM, and complement proteins.

### Treatment protocol for childhood NS

This study was approved by the ethics committee of our institution. Informed consent for renal biopsy was obtained from all the parents, or legal guardians of all patients enrolled in the study. All procedures involving human participants were performed in accordance with the ethical standards of our institutional research committee and the 1964 Declaration of Helsinki and its later amendments.

Children with new-onset NS were empirically treated with oral corticosteroids, using a tapered regimen. Corticosteroids were administered at an initial standard dose of 2 mg/kg body weight twice daily for 4 to 8 weeks, followed by a reduced dose of 1 mg/kg body weight daily for 4 weeks. Finally, the dose was reduced to 2.5 to 5 mg once every 2 to 4 weeks. All patients received angiotensin receptor blockers, such as valsartan (1 mg/kg body weight daily), as adjunctive agents. The doses of these medications were titrated to control proteinuria and blood pressure. The renal function and serum potassium levels were closely monitored.

Second-line immunosuppressive therapy was administered to the children with refractory NS (SRNS, SDNS, and FR-SSNS). The children were assigned to different treatment groups at the discretion of the treating physician. Each group received a different second-line immunosuppressive therapy, per the following protocol: CYC at a dose of 8 to 12 mg/kg body weight for 2 consecutive days every 2 weeks, with a total dose of <150 mg/kg body weight; MMF at a total dose of 15 to 30 mg/kg body weight divided into 2 doses with an interval of 12 hours; TAC at a total dose of 0.05 to 0.15 mg/kg body weight divided into 2 doses with an interval of 12 hours, which was adjusted to maintain TAC trough blood levels at 2.5 to 10 ng/mL.

### Statistical analysis

Measurement data are expressed as the mean (SD). One-way ANOVA, *t* test, and Pearson χ^2^ test were used to compare the demographic data between the CYC, MMF, and TAC groups. The primary outcome variable was the number of patients who achieved complete or partial remission. The secondary outcome variable was the maintenance of remission after discontinuation of treatment. Statistical analyses were performed using the SPSS software version 19.0 (IBM-SPSS, Inc, Armonk, NY). Statistical significance was set at *P* < 0.05.

## Results

### Patient characteristics

A total of 56 children with biopsy-proven MCNS were enrolled in this study. Of the enrolled participants, 83.9% were boys. The follow-up duration ranged from 9 months to 90 months. The children were divided into the CYC (n = 24), MMF (n = 20), and TAC (n = 12) groups based on the immunosuppressive agent used for the treatment of MCNS. The percentage of boys in the CYC, MMF, and TAC groups was 83.3%, 85.0%, and 83.3%, respectively. The age at the time of diagnosis in the CYC, MMF, and TAC groups was 4.3 (2.9) years, 5.2 (2.8) years, and 4.8 (2.3) years, respectively. The level of cystatin C in the CYC, MMF, and TAC groups was 1.1 (0.5) mg/L, 1.0 (0.4) mg/L, and 0.9 (0.3) mg/L, respectively. The demographic and clinical characteristics of the study population are summarized in Table 1. There were no significant differences in age, sex, or cystatin C levels between the 3 groups. The levels of IgG, IgA, IgM, and IgE and the percentages of T cell markers CD3, CD4, CD8, natural killer cell (NK cell) marker CD16^+^CD56^+^, and B cell marker CD19 at the onset of NS were analyzed, and no significant differences were observed among the three groups (Table 1).

**Table 1:** General information and response to second-line immunosuppressive treatment.*

Characteristic	CYC group (n = 24)	MMF group (n = 20)	TAC group (n = 12)	*P* value
Demographic				
Age at diagnosis, y	4.3 (2.9)	5.2 (2.8)	4.8 (2.3)	0.531
Boys†	20 (83.3)	17 (85.0)	10 (83.3)	0.987
Cys-c, mg/L	1.1 (0.4)	1.0 (0.4)	0.9 (0.3)	0.480
FR-SSNS/SDNS†	21 (87.5)	17 (85.0)	10 (83.3)	0.904
Various measures of immune status at the onset of nephrotic syndrome				
IgG, g/L	2.7 (1.9)	2.9 (2.6)	3.6 (2.4)	0.654
IgA, g/L	1.1 (0.6)	1.2 (0.7)	1.1 (0.5)	0.834
IgM, g/L	1.5 (0.6)	1.3 (0.6)	1.5 (0.6)	0.748
IgE, g/L	794.8 (1651.7)	1384.6 (1367.6)	1474.1 (2087.7)	0.829
CD3, %	69.7(12.3)	60.9 (12.4)	69.7 (10.5)	0.209
CD4, %	32.5 (9.5)	29.7 (7.6)	34.5 (10.1)	0.518
CD8, %	29.5 (8.2)	25.8 (12.9)	29.4 (9.2))	0.669
CD4/CD8	1.0 (0.7)	0.7 (0.9)	1.0 (0.9)	0.713
CD56, %	10.8 (6.2)	14.7 (8.8)	10.4 (8.3)	0.370
CD19, %	19.5 (9.6)	22.5 (6.8)	18.8 (5.9)	0.545
Immunofluorescence deposition†				
IgM	17 (70.8)	9 (45.0)	6 (50)	0.199
IgG + A + M + complement	1 (42)	3 (15.0)	1 (8.3)	0.460
IgG + A + M	2 (8.3)	1 (5.0)	1 (8.3)	0.900
Complement	6 (25.0)	6 (30.0)	7 (58.3)	0.128
No immune complex	3 (12.5)	5 (25.0)	1 (8.3)	0.385
Response to treatment†				
Complete remission	17 (70.8)	15 (75.0)	7 (58.3)	0.224
Partial remission	5 (20.8)	5 (25.0)	4 (33.3)	0.351
No remission	2 (8.3)	0	1 (8.3)	–
Steroid-free remission	6 (25.0)	11 (55.0)	9 (75.0)	0.012
Relapse	0 (0)	6 (54.5)	6 (66.7)	0.035
Duration of relapse, mo	0	3.3 (6.9)	3.3 (4.3)	0.975
Steroid dose on relapse, mg/kg	0.5 (0.4)	0.3 (0.3)	0.3 (0.1)	0.443
Immunosuppressants, mo	7.8 (6.6)	20.5 (8.5)	13.6 (17.2)	0.00
Follow-up, mo	27.1 (22.9)	30.1 (25.9)	27.2 (17.2)	0.899

CYC =cyclophosphamide; Cys-c = cystatin C, FR-SSNS = frequent-relapse steroid-sensitive nephrotic syndrome; IG = immunoglobulin; MMF = mycophenolate mofetil; SDNS = steroid-dependent nephrotic syndrome; TAC = tacrolimus.

* Values are presented as mean (SD).

† Values are presented as n (%).

Of the 56 children, 8 (14.3%) had SRNS and 48 (85.7%) had FR-SSNS/SDNS. There were 21 (87.5%), 17 (85.0%), and 10 (83.3%) children with FR-SSNS/SDNS in the CYC, MMF, and TAC groups, respectively. Pathological evaluation of renal biopsies revealed MCNS in all children. The intensity of immunofluorescence staining of pathological specimens obtained from the three groups was compared, and no significant differences were observed (Table 1).

### Response to CYC therapy

A total of 21 children with FR-SSNS/SDNS and 3 children with SRNS were treated with CYC. Complete remission was achieved in 17 (70.8%) patients, partial remission in 5 (20.8%) patients, and no remission in 2 (8.3%) patients. The mean follow-up duration of children treated with CYC was 32.6 (21.2) months. Six of the 24 patients (25%) achieved steroid-free remission at the last follow-up. None of the patients developed relapse after discontinuation of medication.

### Response to MMF therapy

Overall, 17 children with FR-SSNS/SDNS and 3 children with SRNS underwent a ≥1 MMF trial. Complete remission was achieved in 15 (75.0%) patients, partial remission in 5 (25.0%) patients, and none of the patients were nonresponders. The combined rate of complete and partial response to MMF was 100%. The mean follow-up duration in the MMF group was 43.0 (19.7) months. At the final follow-up, 11 (55%) of the 15 patients who achieved complete remission had remission after discontinuation of their medications. Among the 11 patients who achieved remission off medications, 6 (54.6%) developed a relapse, and the mean duration of relapse after discontinuation of medications was 3.3 (6.9) months. The level of CD16^+^CD56^+^ expression was significantly higher in children with relapse than in those without relapse (*P* = 0.042) (Table 2).

**Table 2:** Various measures of immune status between steroid-free or not-free remission in 3 treatment groups.*

Steroid-free remissions	CYC (n = 24)		MMF (n = 20)		TAC (n = 12)	
	Yes (n = 6)	No (n = 18)	Yes (n = 11)	No (n = 9)	Yes (n = 9)	No (n = 3)
IgG, g/L	1.6 (1.5)	3.0 (2.0)	3.6 (2.6)	2.4 (2.8)	4.2 (2.5)	1.8 (0.3)†
IgA, g/L	0.8 (0.8)	1.2 (0.5)	1.4 (0.5)	1.0 (1.0)	1.2 (0.5)	0.8 (0.5)
IgM, g/L	1.3 (0.8)	1.6 (0.5)	1.4 (0.5)	1.1 (0.7)	1.5 (0.6)	1.5 (0.4)
IgE, g/L	704.8 (1359.5)	822.9 (1772.3)	1427.7 (1434.2)	1320.0 (1391.9)	1535.9 (2350.8)	1288.6 (1340.4)
CD3, %	68.3 (15.9)	70.2 (11.3)	61.6 (13.9)	59.9 (11.8)	68.9 (12.7)	71.5 (5.8)
CD4, %	33.8 (11.1)	32.0 (9.3)	31.9 (9.5)	26.4 (1.0)	33.8 (12.5)	35.9 (3.6)
CD8, %	29.7 (10.6)	29.4 (7.7)	25.5 (15.9)	26.1 (8.7)	29.0 (10.8)	30.3 (6.9)
CD4/CD8	1.3 (0.8)	0.8 (0.7)	0.9 (1.1)	0.5 (0.7)	1.0 (1.1)	1.3 (0.5)
CD56, %	10.6 (5.4)	10.9 (6.6)	15.4 (10.8)	13.6 (5.8)	13.1 (9.0)	4.9 (3.1)
CD19, %	23.6 (15.0)	18.1 (7.6)	21.8 (5.9)	23.7 (8.9)	17.3 (5.6)	21.8 (6..6)

CYC =cyclophosphamide; IG = immunoglobulin; MMF = mycophenolate mofetil; TAC = tacrolimus.

* Values are presented as mean (SD).

† *P* = 0.017.

### Response to TAC therapy

Overall, 10 children with SSNS/SDNS and 2 children with SRNS underwent ≥1 TAC trial. Complete remission was achieved in 7 (58.3%) patients, partial remission in 4 (33.3%) patients, and no remission in 1 (8.3%) patient. The combined rate of complete and partial response to TAC was 91.7%. The mean duration of TAC therapy was 13.6 (3.9) months and the mean follow-up was 32.6 (12.8) months. Among the patients who achieved complete or partial remission, 9 (75%) had sustained remission after discontinuation of medications at the final follow-up. Among the patients who achieved remission after discontinuation of medications, 6 developed a relapse, and the mean duration of relapse posttreatment cessation was 3.3 (4.3) months. In the TAC group, the IgG level at the onset of NS was significantly higher in children who achieved remission after discontinuation of medication at the final follow-up than in those who continued TAC therapy (*P* = 0.017) (Table 3).

**Table 3:** Various measures of immune status between with and without relapse in mycophenolate mofetil (MMF) and tacrolimus (TAC).*

	MMF (n = 11)		TAC (n = 9)	
	Relapse (n = 6)	No relapse (n = 5)	Relapse (n = 6)	No relapse (n = 3)
IgG, g/L	4.1 (3.1)	2.0 (0.6)	4.5 (2.9)	3.7 (1.5)
IgA, g/L	1.3 (0.4)	1.4 (0.6)	1.8 (0.5)	1.3 (0.3)
IgM, g/L	1.5 (0.6)	1.2 (0.4)	1.6 (0.7)	1.5 (0.5)
IgE, g/L	121.2 (1486.1)	1695.8 (1538.8)	919.1 (1467.9)	2769.4 (3646.2)
CD3, %	55.1 (11.4)	74.8 (7.6)	69.1 (18.2)	68.7 (8.3)
CD4, %	32.8 (10.6)	30.1 (9.9)	39.0 (7.4)	28.6 (15.9)
CD8, %	17.7 (5.7)	41.2 (20.8)	23.6 (10.1)	34.3 (10.1)
CD4/CD8	2.0 (1.0)	0.9 (0.7)	1.9 (1.0)	1.0 (0.9)
CD56, %	21.2 (7.2)	3.8 (5.4)†	13.8 (13.2)	12.4 (5.1)
CD19, %	23.2 (6.8)	18.93 (3.0)	16.3 (7.6)	18.2 (4.0)

IG = immunoglobulin.

* Values are presented as mean (SD).

† *P* = 0.042.

## Discussion

In this study, CYC, MMF, and TAC were administered as second-line therapies for the treatment of childhood refractory MCNS. The demographic and clinical characteristics of the 3 groups did not differ significantly. We found no significant differences in the proportion of immunofluorescence deposition in the renal tissues among the 3 groups. The response and relapse rates after treatment discontinuation were higher in the TAC group compared with those in the MMF and CYC groups. The response and relapse rates after treatment cessation were the lowest in the CYC group.

The treatment of SRNS, SDNS, and FR-SSNS in pediatric patients remains a challenge because the guidelines for treatment procedures, and studies on expected treatment outcomes, remain limited. NS has been reported to be associated with changes in the immune response.^16^ A previous study has reported that the pathological patterns of renal biopsies assessed by immunofluorescence are related to the therapeutic effects of corticosteroids in pediatric NS.^17^ The clinical guidelines for the treatment of FR-SSNS recommend the use of alkylating agents such as CYC and chlorambucil as corticosteroid-sparing agents, based on moderate quality of evidence and those for the treatment of SDNS suggest the use of alkylating agents based on low quality of evidence.^18^ The rate of steroid-free remission in the CYC group was 25%, which is similar to the percentage of patients (30%–35%) with minimal change in disease attaining long-term remission after CYC treatment.^19^

Mycophenolic acid, the active metabolite of MMF, is a selective, reversible inhibitor of inosine monophosphate dehydrogenase, which inhibits the de novo synthesis of purines. Thus, it exerts a cytostatic effect specifically on T and B lymphocytes and is effective in treating refractory MCNS, including SRNS and SDNS.^20^ In this study, the combined rate of complete and partial response to MMF was 100%. Our findings suggest that MMF is an adequately potent agent for treating proteinuria in both SDNS and SRNS. However, the steroid-free remission and relapse rates after treatment discontinuation in the MMF group were 55% and 54.5%, respectively. Recent studies have reported that mycophenolic acid at a dose of more than 45 mg/h/L might be more effective in reducing the rates of relapse.^21^ Unfortunately, we did not monitor mycophenolic acid concentrations. Notably, the proportion of CD16^+^CD56^+^-positive cells in children with MCNS relapse was much higher than that in children without relapse (21.2% [7.2%] vs 3.8% [5.4%]; *P* = 0.042). The subgroups of NK cells in NS patients will change before and after remission. An increase in CD56^high^ NK cells and decrease in CD56^low^ NK cells occurred significantly at the onset of SSNS. NK cells may be involved in the pathogenesis of NS.^12^^,^^22^

A recent addition to the armamentarium of therapies for SRNS is a macrolide immunosuppressant, TAC, which inhibits calcineurin and blocks the nuclear translocation of the cytosolic component of the nuclear factor of activated T cells. Genes regulated by the transcription factor of nuclear factor of activated T cells are essential for T cell proliferation and the expression of the prototypic T cell growth factor interleukin 2. Previous studies on TAC have reported similar outcomes to those of our study in patients with SRNS; however, there are no studies on its long-term adverse effects. Nonetheless, the current study showed satisfactory outcomes for children with SRNS. In this study, the combined rate of complete and partial response to TAC was 91.7%. Although the relapse rate after discontinuation of medication was 66.7%, maximal steroid-free remission was achieved in all 3 groups. CYC and MMF are less effective than TAC in inducing sustained remission in children with idiopathic SRNS.^23^^,^^24^ TAC trough concentration in the range of 2.1 to 4.8 ng/mL has been recommended in children with SRNS.^25^ The trough concentrations of TAC in this study were >2.5 ng/mL. Children in whom steroid therapy was discontinued had significantly higher IgG levels at the onset of NS compared with those in whom steroid treatment was continued. A high IgG level might thus be a favorable prognostic factor in patients with MCNS undergoing TAC treatment.^13^^,^^14^

This study has a few limitations. The study was conducted at a tertiary center, and the outcomes were objectively measured by professional physicians. Nevertheless, owing to the retrospective design of the study, we cannot make a causal conclusion. Additionally, selection bias for a single-center study with a small sample size and measurement bias might not be excluded. It is possible that residual confounders, such as socioeconomic factors, exist, which might introduce study bias. Considering the maximum cumulative dose of CYC, the duration of CYC therapy in this study was short. Due to the small sample size and single-center study design, the generalization of our conclusions might be limited. Our findings warrant further study with the need for a well-designed, large-scale, prospective study.

## Conclusions

For the treatment of refractory MCNS in children, the relapse rate following TAC therapy was higher than that following MMF and CYC therapy. Different proportions of CD16^+^CD56^+^-positive cells may be associated with relapse rates in patients with MCNS receiving MMF treatment.

## Conflicts of Interest

The authors have indicated that they have no conflicts of interest regarding the content of this article.
